# A 15-year experience highlighting the spectrum of Alport kidney disease in the pediatric population and novel genetic variants in *COL4A3–5*

**DOI:** 10.1007/s00467-025-06683-8

**Published:** 2025-02-05

**Authors:** Nastja Andrejašič, Anja Blejc Novak, Mirjam Močnik, Nataša Marčun Varda, Špela Stangler Herodež, Danijela Krgović, Andrej Zupan, Anamarija Meglič

**Affiliations:** 1 Department of Pediatrics, General Hospital Izola, Polje 40, Izola, Slovenia; 2 The Faculty of Medicine University of Ljubljana, Vrazov trg 2, 1000 Ljubljana, Slovenia; 3 Department of Pediatrics, University Medical Centre Maribor, Ljubljanska ulica 5, 2000 Maribor, Slovenia; 4 Clinical Institute for Genetic Diagnostics, University Medical Centre Maribor, Ljubljanska ulica 5, 2000 Maribor, Slovenia; 5 Institute of Pathology, Faculty of Medicine, University of Ljubljana, Korytkova 2, 1000 Ljubljana, Slovenia

**Keywords:** Alport kidney disease, Children, Phenotype, Genotype, *COL4A3–5*

## Abstract

**Background:**

Alport kidney disease (AKD) presents one of the most prevalent genetic kidney disorders, characterized by a complex genetic background and diverse clinical manifestations. This study aimed to review the clinical and genetic features of pediatric patients with *COL4A3–5* variants and identify novel genetic variants.

**Methods:**

Data were collected retrospectively at a national level from pediatric patients up to 19 years old, who underwent genetic testing between 2008 and 2023. Patients with pathogenic and likely pathogenic *COL4A3–5* variants were included. Their clinical, laboratory, and genetic characteristics were presented.

**Results:**

Over 15 years, 85 children and adolescents tested positive for pathogenic or likely pathogenic *COL4A3–5* variants. Increasing incidence was noted as genetic testing became more prevalent. One patient (1.2%) progressed to kidney failure and six (7%) had extrarenal involvement. Pathogenic or likely pathogenic variants in *COL4A3*, *COL4A4*, and *COL4A5* genes were found in 14 (16.4%), 34 (40.0%), and 37 (43.6%) patients, respectively. Patients were diagnosed with autosomal, X-linked, and digenic AKD in 55.2%, 43.6%, and 1.2%, respectively. Eight novel variants were recorded, and their associated phenotype presented.

**Conclusions:**

This study expands the genetic and clinical background of pediatric patients with AKD, presenting on a spectrum from mild hematuria to progressive chronic kidney disease. Genetic confirmation and risk stratification in the pediatric population are critical to ensure timely care and potentially slow down the progression of kidney disease.

**Graphical abstract:**

**Figure:**
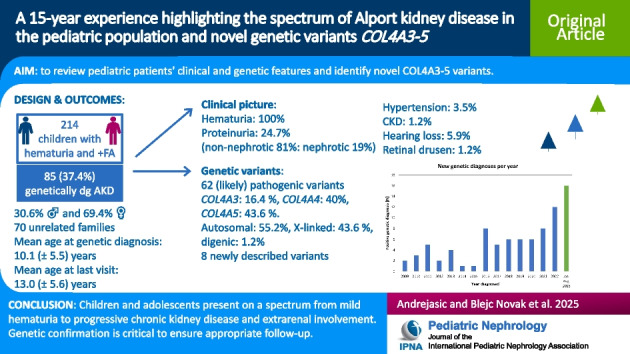
A higher resolution version of the Graphical abstract is available as Supplementary information

**Supplementary Information:**

The online version contains supplementary material available at 10.1007/s00467-025-06683-8.

## Introduction

Alport kidney disease (AKD) is a spectrum of kidney disorders caused by pathogenic variants in the α3, α4, and α5 chains of collagen type IV, which are translated from the *COL4A3*, *COL4A4*, and *COL4A5* genes, respectively. Collagen type IV is the major structural component of the glomerular basement membrane (GBM) and the basement membranes of the inner ear and eye, resulting in their dysfunction when pathogenic variants in the aforementioned genes occur [1, 2]. Pathogenic variants in collagen type IV genes are the most frequent cause of chronic kidney disease (CKD) of unknown etiology and one of the most common causes of inherited kidney disease [3, 4].

In the era of the genetic revolution, genetic testing is now prioritized over kidney biopsy for the diagnosis of AKD [5–7]. Children and adolescents with pathogenic (P) and likely pathogenic (LP) variants in any of the *COL4A3*–*5* genes can have a wide range of phenotypes, from mild microhematuria to nephrotic proteinuria and progressive CKD. Extrarenal manifestations are also possible, such as sensorineural hearing loss and ocular abnormalities, less commonly vascular abnormalities [8, 9]. With the increasing use of molecular genetic testing in clinical practice, pathogenic variants in *COL4A3*–*5* genes have been increasingly reported in patients with diverse clinical presentations, such as proteinuria-predominant phenotype, kidney failure of unknown etiology, familial immunoglobulin A (IgA) nephropathy with thin GBM, and kidney cystic disease with negative genetic testing for polycystic kidney diseases [8].

Therefore, these findings challenge the traditional classification of Alport syndrome (AS) which includes childhood-onset hematuria, followed by proteinuria and progressive decline in kidney function during adolescence and young adulthood, along with sensorineural hearing loss and eye abnormalities [8]. Newer terms have been used in the literature to denote the kidney disease states linked to pathogenic variants in *COL4A3–5* genes. In 2018, Kashtan et al. published a position paper of the Alport Syndrome Classification Working Group with the aim to improve kidney outcomes through regular monitoring and early treatment, a unified classification of genetic disorders of the collagen IV α345 triplet helical molecules [10]. Clinical practice recommendations for the diagnosis and management were then published in 2020 and an update in 2021 [7]. The latest guidelines for genetic testing and management of AS were published in 2022 [12]. In 2024, Puapatanakul et al. distinguished “classic” AS from the less severe nonsyndromic genetically related nephropathies and suggested calling them AKD [8]. Further research and discussion are needed to determine which nomenclature and classification most effectively encompasses all patients. In our study, the term AKD was used whenever a P/LP variant in the *COL4A3–5* genes was detected in patients exhibiting signs of kidney disease.

Pathogenic variants in *COL4A5* are associated with X-linked inheritance. Until recently, X-linked AS (XLAS) accounted for approximately two-thirds of AS [5, 11]. Autosomal recessive AS (ARAS) has been defined by biallelic pathogenic variants in *COL4A3* or *COL4A4.* Individuals with a pathogenic heterozygous *COL4A3* or *COL4A4* variant are diagnosed as a carrier state of ARAS, autosomal dominant AS (ADAS), or thin basement membrane nephropathy. Digenic inheritance is applied with the occurrence of two variants, each in a different gene [10].

Our nationwide study aimed to review the data, genetic evaluations, and phenotypic spectrum of children and adolescents with AKD in our country over the longest possible period. We focused on a period of 15 years during which these patients underwent regular genetic diagnostics.

## Methods

### Patients and data collection

In Slovenia, only two centers, University Medical Center Ljubljana and University Medical Center Maribor, monitor pediatric patients with AKD and both were included in the study, which represents data at the national level. Children and adolescents up to 19 years old who were referred to genetic testing between February 2008 and March 2023 because of persistent hematuria and/or a positive family history of kidney disease were included in the study. In the next step, patients without a genetically confirmed AKD diagnosis were excluded from further analysis. Written informed consent for the genetic analysis was obtained from all patients or their parents/caregivers before the analysis. All data were collected retrospectively, and all information was kept anonymous. Patients’ clinical manifestations, family history, laboratory characteristics at first presentation, genetic test results, kidney biopsy results (if available), and last follow-up visit characteristics were collected from medical records for analysis. The Commission of the Republic of Slovenia on Medical Ethics (0120–127/2024–2711-4) and the Institutional Ethics Committee in Maribor (protocol code UKC-MB-KME-4/24) approved the study. The study was performed in accordance with the Helsinki Declaration.

### Definitions

AKD was diagnosed based on the discovery of pathogenic (P) or likely pathogenic (LP) variants in the *COL4A3–5* genes, using genetic analysis. A variant of unknown significance (VUS) was classified and reported according to current genetic testing guidelines [6, 12].

Autosomal AKD includes patients with P/LP variants in either *COL4A3* or *COL4A4* genes. Patients with a single P/LP variant were classified as heterozygotes, while those with two different P/LP variants in trans were classified as compound heterozygotes. Patients with two identical P/LP variants in trans were classified as homozygotes. Patients with two P/LP variants in different genes were classified as digenic heterozygotes. Females with one P/LP variant in *COL4A5* were classified as heterozygotes and males as hemizygotes. A VUS does not affect zygosity [6, 12].

Proteinuria was defined as a spot urine protein/creatinine ratio greater than 20 mg/mmol or a 24-h urine protein concentration greater than 4 mg/m^2^/h. Nephrotic proteinuria was defined as a spot urine protein/creatinine ratio greater than 200 mg/mmol or a 24-h urine protein concentration greater than 40 mg/m^2^/h.

Glomerular filtration rate (GFR) was calculated using Schwartz’s original formula for patients up to 18 years old or the CKD-EPI formula for patients older than 18 years [13]. When defining CKD, a GFR of 60 mL/min/1.73 m^2^ or less was used.

### Genetic testing and variant interpretation

Panel testing for hereditary causes of hematuria was performed at the Institute of Pathology, Faculty of Medicine, University of Ljubljana and the Laboratory of Genomics at the Clinical Institute for Genetic Diagnostics, University Medical Centre Maribor. Due to the long timespan, two different strategies were used for genetic testing. The first was the single-stranded conformation polymorphism (SSCP) method in conjunction with Sanger sequencing, as previously described [14], and the second was targeted NGS panel sequencing, as previously described [15]. Patients with negative SSCP genetic result were retested using NGS in cases of a high level of suspicion of disease (a positive family history of AS, development of significant proteinuria, or histologic diagnosis of AS).

### Statistical analysis

Categorical variables were expressed as frequencies and percentages. Continuous variables were expressed as the mean ± standard deviation (mean ± SD) or the median and interquartile range in case of skewed distributions. The follow-up time was determined as the number of months from genetic analysis to the last clinical visit.

The analysis was performed using IBM® SPSS® Statistics 22 (International Business Machines Corp., Armonk, NY) for Windows.

## Results

### Patient characteristics and follow-up

In the timeframe from February 2008 to March 2023, samples of 214 pediatric patients were sent for genetic analysis due to persistent hematuria and/or a positive family history of kidney disease (hematuria, AKD, CKD, hearing loss). There were 80 (37.4 %) samples from the University Children’s Hospital Ljubljana and 134 (62.6 %) from the University Medical Centre Maribor, Department of Pediatrics. The patients were aged 0 to 19 years (mean age 10.5 ± 5.5 years), 89 (41.6%) were male, and 125 (58.4%) were female. Eighty-five (37.4%) samples were positive for at least one P/LP variant in the genes *COL4A3*–*5* and 129 samples were negative. Genetic diagnosis without a history of hematuria was performed only in two patients, where genetic diagnosis was established prenatally due to a positive family history of AKD.

Of the 85 children with a positive genetic diagnosis, seven (8.2%) had a kidney biopsy due to persistent hematuria and proteinuria before the genetic diagnosis was confirmed. In three patients, genetic testing was initially negative using SSCP, but later turned positive using NGS. Five patients had a positive family history of CKD or hematuria and two had a negative family history.

Among the 129 patients with negative genetic diagnosis, four patients (3.1%) had a kidney biopsy positive for AKD. All of these were genetically tested using NGS and no LP/P variants or VUS in *COL4A3–5* were detected. Two had a negative and two had a positive family history of hematuria or CKD. In one of them, kidney failure occurred by the age of 14 years, with eye and inner ear involvement.

**Fig. 1:**
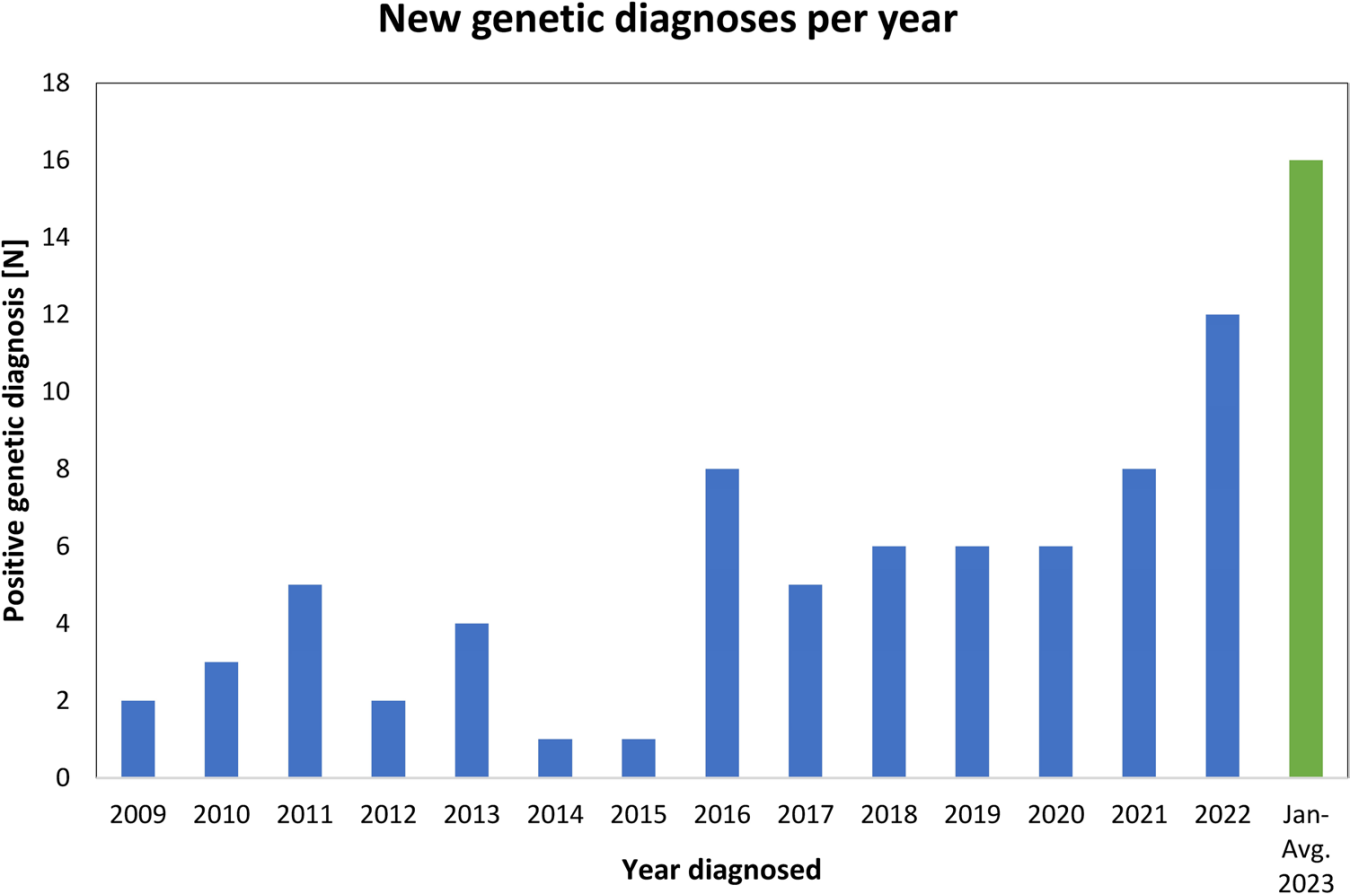
New genetic diagnoses during the years (*N*, number). The number of positive results in the first 8 months of 2023 implies further growth

The first results to include patient’s genetic analysis dated to 2009. The number of genetic diagnoses of AKD grew over the years (Fig. 1). Among 85 genetically diagnosed AKD patients from 70 unrelated families, 26 (30.6%) were male, and 59 (69.4%) were female. The mean age at the time of genetic diagnosis was 10.1 (± 5.5) years.

The median time from the first visit to genetic diagnosis was 10 (range 11 to 186) months. There were two patients genetically diagnosed prenatally and two patients, who had a genetic diagnosis confirmed before their first clinical nephrological visit due to the evaluation of an older sibling. The last date for follow-up evaluation was 31^st^ August 2023. Patients were followed up until the last clinical visit in this timeframe. The mean follow-up time from genetic diagnosis to the last visit was 35 months (range 0–165) months. There were 10 (11.8%) AKD patients with only one visit during the time of our study.

At the last follow-up visit, mean age was 13.0 (± 5.6) years. All patients had hematuria during the follow-up. Proteinuria was found in 21 (24.7%) patients at the last visit, four (19.0%) of which had nephrotic range proteinuria. Other causes of nephrotic proteinuria were excluded and kidney biopsy changes were specific to AKD. There were three (3.5%) patients with hypertension and one (1.2%) patient with CKD since the age of 15 years. Six (7%) patients had extrarenal involvement, five (5.9%) had sensory hearing loss confirmed at the ages of 7 to 18 years, and one (1.2%) in whom retinal drusen were confirmed at the age of 10 years (Table 1).

**Table 1:** Characteristics of patients with extrarenal involvement

Organ involved	Variant	Age (years)*	Gender
Ear	*COL4A3*: c.1006G>T (p.Gly336Cys)	8	M
Ear	*COL4A3*: c.1883G>A (p. Gly28Asp)	13	F
Ear	*COL4A4*: c.117_285+3del (p.Pro441_Ser456del)	12	M
Ear	*COL4A5*: c.1871G>A (p.Gly624Asp)	18	M
Ear	*COL4A5*: c.796C>T (p.Arg266Ter)	7	M
Eye	*COL4A5*: c.1871G>A (p.Gly624Asp)	10	M

### Genetic variant characteristics and phenotype correlations

A total of 69 different variants were identified (62 P/LP and 7 VUS) in 85 patients. In 9 (10.6%) patients, two different variants were identified. The P/LP variants in *COL4A3*, *COL4A4*, and *COL4A5* genes were identified in 14 (16.4%), 34 (40.0%), and 37 (43.6%) patients, respectively. Patients were diagnosed with autosomal, X-linked, and digenic AKD in 47 (55.2%), 37 (43.6%), and one (1.2%), respectively. The distribution of affected genes associated with the type of inheritance and clinical picture at the last visit are presented in Table 2.

**Table 2:** Variability of affected genes and clinical picture at the last visit

Genes affected	*COL4A3*	*COL4A3+A3*	*COL4A3+A4*	*COL4A4*	*COL4A4+A3*	*COL4A4+A4*	*COL4A4+A5*	*COL4A5*	*COL4A5+A3*
**Patients**: *N* (%)	11 (12.9)	2 (2.4)	1 (1. 2)	29 (34.1)	3 (3.5)	1 (1.2)	1 (1.2)	36 (42.4)	1 (1.2)
**Type of inheritance**	Autosomal	Autosomal	Digenic	Autosomal	Autosomal*	Autosomal*	Autosomal*	X-linked	X-linked*
**Zygosity**	Heterozygote	Compound heterozygote	Digenic heterozygote	Heterozygote	Heterozygote*	Heterozygote*	Heterozygote*	Hemi-/heterozygote	Heterozygote*
**Age at diagnosis**: mean ± SD (years)	12.1±3.8	15.9±3.1	14.8	10.4±4.5	13.7±3.6	18.9	16.9	7.8±5.9	19.8
**Age at last visit**: mean ± SD (years)	13.7±4.6	18.6±2.3	14.8	11.7±4.7	19.0±3.4	18.9	17.9	12.4±6.4	19.8
**Gender**: M/F (*N* (%))	5/6 (45/55)	1/1 (50/50)	0/1 (0/100)	10/19 (35/65)	1/2 (33/67)	0/1 (0/100)	0/1 (0/100)	9/27 (25/75)	0/1 (0/100)
**GFR**: >60/<60 (*N* (%))	11/0 (100/0)	1/1 (50/50)	1/0 (100/0)	29/0 (100/0)	3/0 (100/0)	1/0 (100/0)	1/0 (100/0)	36/0 (100/0)	1/0 (100/0)
**Hematuria**: yes/no (*N* (%))	8/3 (73/27)	2/0 (100/0)	1/0 (100/0)	25/4 (86/14)	2/1 (67/33)	0/1 (0/100)	1/0 (100/0)	34/2 (94/6)	1/0 (100/0)
**Proteinuria**: yes/no (*N* (%))	3/8 (27/73)	2/0 (100/0)	0/1 (0/100)	4/25 (86/14)	2/1 (67/33)	0/1 (0/100)	0/1 (0/100)	10/26 (28/72)	0/1 (0/100)
**Ear**: involved/not/A (*N* (%))	2/3/6 (18/27/55)	0/2/0 (0/100/0)	0/0/1 (0/0/100)	1/5/23 (4/17/79)	0/2/1 (0/67/33)	0/0/1 (0/0/100)	0/0/1 (0/0/100)	2/23/11 (6/64/30)	0/1/0 (0/100/0)
**Eye**: involved/not/A (*N* (%))	0/5/6 (0/45/55)	0/2/0 (0/100/0)	0/0/1 (0/0/100)	0/5/24 (0/17/83)	0/2/1 (0/67/33)	0/0/1 (0/0/100)	0/0/1 (0/0/100)	1/22/13 (3/61/36)	0/1/0 (0/100/0)

*N*, number; *M*, male; *F*, female; *GFR*, glomerular filtration rate in mL/min/1.73 m^2^; *A*, awaiting assessment; *a VUS was identified in the second gene

The clinical picture of patients with the four most common P/LP variants in our cohort is summarized in Table 3. The most commonly encountered variant was *COL4A5: c.1871G>A (p.Gly624Asp)*, found in nine patients from six unrelated families. Among these patients, five (56%) were male and four (44%) were female, aged from 4 to 18 years at the time of diagnosis. All had normal GFR. Except for three cases, the clinical picture was isolated microhematuria. The three exceptions were a male who presented also with retinal drusen at the age of 10 years and had no hematuria at the last clinical visit, a male with additional nephrotic range proteinuria since the age of 16 years, which persisted at the age of 19 years despite treatment with the maximal dose of angiotensin-converting enzyme inhibitors (ACEI), and a male with confirmed additional sensory hearing loss.

**Table 3:** Clinical picture of patients with the four most common P/LP variants at their last clinic evaluation

Variant	Patients/families (*N*)	Age at diagnosis range (years)	Age at last visit range (years)	Gender M/F (*N* (%))	GFR >60/<60 (*N* (%))	Hematuria yes/no (*N* (%))	Proteinuria yes/no (*N* (%))	Ear involved/not/A (*N* (%))	Eye involved/not/A (*N* (%))
*COL4A5:* c.1871G>A (p.Gly624Asp)	9/6	4.0–18.0	4.0–21.0	5/4 (56/44)	9/0 (100/0)	8/1 (89/11)	1/8 (11/89)	1/4/4 (11/44.5/44.5)	1/3/5 (11/33/56)
*COL4A5*: c.81+1G>A (p.?)	3/2	2.0–13.5	6.0–17.0	0/3 (0/100)	3/0 (100/0)	3/0 (100/0)	2/1 (67/33)	0/3/0 (0/100/0)	0/3/0 (0/100/0)
*COL4A5:* c.1598G>A (p.Gly533Glu)	3/2	0–16.3	1.5–16.3	0/3 (0/100)	3/0 (100/0)	2/1 (67/33)	0/3 (0/100)	0/2/1 (0/67/33)	0/1/2 (0/33/67)
*COL4A4*: c.3044G>A (p.Gly1015Glu)	3/2	3.6–6.8	3.6–7.7	0/3 (0/100)	3/0 (100/0)	2/1 (67/33)	0/3 (0/100)	0/0/3 (0/0/100)	0/0/3 (0/0/100)

*M*, male; *F*, female; *GFR*, glomerular filtration rate in mL/min/1.73 m^2^; *A*, awaiting assessment

In the study, eight novel variants were identified, which are presented in Table 4 along with their clinical presentation at the last visit.

**Table 4:** Clinical picture of patients with variants that have previously not been described in the literature. The pathogenicity was evaluated according to the American College of Molecular Genetics (ACMG) guidelines

	Newly described variant and pathogenicity	Additional variant	Gender	Age at last visit	GFR	Hematuria	Proteinuria	Ear involved	Eye involved
1	**VUS:** *COL4A3*: c.2609T>C (p.Leu870Pro)	*COL4A4*: c.3743G>A (p.Gly1248Glu)	M	15 y	>90	Since 14 y	Since 14 y	No	No
2	**Pathogenic:** *COL4A4*: c.193G>C (p.Gly65Arg)	/	F	17.3 y	>90	Since 3 y	No	No	No
3	**VUS:** *COL4A5*: c.3245A>G (p.Lys1082Arg)	/	F	23 y	>90	Since 12 y	No	No	No
4	**Likely pathogenic:** *COL4A4*: c.3682A>T (p.Lys1228Ter)	/	F	4.8 y	>90	Since 3 y	No	No	No
5	**Likely pathogenic:** *COL4A5*: c.1700 G>A (p. Gly567Glu)	/	F	6 y	>90	Since 2 y	No	No	No
6	**Pathogenic:** *COL4A5*: c.321+1G>C (p.?)	/	F	1.9 y	>90	Since 1 y	No	A	A
7	**Likely pathogenic:** *COL4A5*: c.4181G>A (p.Gly1394Asp)	*COL4A3*: c.3644G>A (p.Arg1215Gln)*	F	19.8 y	>90	Since 12 y	No	No	No
8	**Likely pathogenic:** *COL4A4*: c.4327G>T (p.Gly1443Ter)	/	F	8 y	>90	Since 3 y	No	No	No

*M*, male; *F*, female; *GFR*, glomerular filtration rate in mL/min/1.73 m^2^; *y*, years; *A*, awaiting assessment

## Discussion

The purpose of this retrospective study was to evaluate the genetic background and clinical picture of pediatric patients with *COL4A3–5* P/LP variants in Slovenia, to add genotype–phenotype correlations to previously published data, and to report novel genetic variants (Tables 1, 2, 3, and 4). The *COL4A5* gene was most frequently affected, but a significant proportion of P/LP variants were also found in the *COL4A3* and *COL4A4* genes, resulting in a high percentage of autosomal AKD. Despite a generally mild clinical presentation, some patients still exhibited a significant manifestation during childhood.

AKD is considered a rare disease in both pediatric and adult nephrology, estimated to occur in about 1:5000–10,000 live births [16]. Since there are about 15,000–20,000 live births per year in our country, we could expect three to four new patients with AKD every year. In the 15 years of our study, we focused on AKD in its broad meaning and found 85 children with P/LP variants in *COL4A3–5* genes, which corresponds to an average of about 5.6 patients per year, which is higher than expected. A recent examination of a large variant database from individuals without known kidney disease, conducted by Gibson et al., predicted pathogenic *COL4A5* variants in at least one in 2320 individuals, and heterozygous pathogenic *COL4A3* or *COL4A4* variants in one in 106 individuals [17]. However, the true prevalence of potentially pathogenic *COL4A3*–*5* variants is likely even higher because the researchers did not account for all possible gene variants and the previously established AKD. Furthermore, the prevalence of the various forms of AKD inheritance is influenced by the penetrance of hematuria and kidney impairment in these variants. Future research should focus on the penetrance of these clinical features [17]. Figure 1 in our study shows a significant increase in the number of genetically confirmed AKD cases in the last 3 years of the study. In the first 8 months of 2023, 16 new cases were confirmed, with the expectation of reaching an even higher number in the years to come. We believe this resulted from the joint efforts of both centers to standardize and expand genetic testing whenever a *COL4A3–5* pathogenic variant is suspected according to the latest guidelines [6, 7, 12]. Additionally, improvement in the accuracy of genetic testing, and shortened turnaround time (from an average of 0.5–3 years until 2012 to an average of 0.2–1 year after 2012) resulted in detecting more and more variants. As the trend of our study suggests, the incidence of AKD may change significantly in the future with the increasing use of genetic testing. Furthermore, new recommendations suggest expanding the spectrum of AKD to include milder, genetically related forms [8], as described and included in our study. With these improvements, it is important to note the declining necessity for kidney biopsies. Most children have a mild clinical phenotype; therefore, it is possible to wait for the result of genetic diagnosis and avoid invasive procedures. A positive genetic diagnosis mostly eliminates the need for a biopsy. In cases of a negative genetic diagnosis, a biopsy is only performed if hematuria is accompanied by proteinuria [4, 7].

Factors such as family history of kidney disease and the presence of hematuria were shown to be strong predictors of a positive genetic diagnosis. In a recent study by Rheault et al., it was found that 28.1% of children with risk factors for genetic kidney disease received a positive genetic diagnosis, with the majority exhibiting variants linked to AKD. Among children meeting both criteria (hematuria and positive family history of CKD), the genetic diagnostic rate increased to 40.4% [18]. Genetic testing in our cohort of patients was performed in cases of unexplained persistent hematuria and a positive family history of kidney disease, and we achieved a genetic diagnosis rate of 40%.

Since all of our included patients were children and adolescents, the clinical presentation was mild, as expected. Hematuria was uniformly present, while proteinuria was observed in almost one-quarter of the cases, consistent with findings from previous studies [5, 8]. Other clinical manifestations, such as sensorineural hearing loss and ocular abnormalities, were rare (Table 2). For more precise genotype–phenotype correlations, we collected data for the four most frequent variants in our cohort in Table 3. In our efforts to establish further genotype–phenotype correlations, we identified two patients with a more severe clinical presentation. The first patient is a female with a *COL4A3*: c.1883G>A (p. Gly28Asp) variant, who presented with nephrotic range proteinuria and a known sensorineural hearing loss at the first nephrological visit. Her variant was classified as a VUS at the time of the diagnosis but was later reclassified to likely pathogenic. The same variant was reported in 2022, in a female patient with microhematuria and nephrotic range proteinuria, whose disease progressed to kidney failure, which is of clinical significance in the management of our patient [19]. The second patient with a more severe clinical presentation is a male compound heterozygote for the *COL4A3* gene*.* Even though our cohort included two compound heterozygotes and nine hemizygotes, only he developed CKD. At the age of 3 years, he presented with microhematuria and episodes of macrohematuria. Proteinuria occurred at the age of 7 years. A biopsy was performed at the age of 11 years and showed changes specific to AS. CKD was diagnosed at the age of 15 years, and at the age of 20 years, he was about to receive a kidney transplant. During our follow-up, he did not develop extrarenal symptoms. The first genetic testing was performed at the age of 3 and was negative. His genetic diagnosis was established at the age of 18, using NGS, and revealed two pathogenic variants *in trans* – *COL4A3*: c.2881+1G>A (p.?) and *COL4A3*: c.4421T>C (p. Leu1474Pro). The first variant was recently described in a population of 34 Croatian patients, among whom 14.7% progressed to kidney failure at the median age of 48 years [20]. Progression occurred significantly earlier in the case of our patient, which could be due to the influence of the second variant. There are conflicting data on the pathogenicity of the *COL4A3*: c.4421T>C (p.Leu1474Pro) variant, which per se might not result in a clinical picture of AKD, but has been reported as pathogenic in combination with other variants [21]. Our patient’s parents, who each have one variant, did not develop proteinuria or kidney failure during our follow-up. Advances in knowledge allow for more accurate prediction of variant-specific manifestations, which, as shown, can have significant clinical implications.

We have identified 69 different genetic variants in the *COL4A3–5* genes. *COL4A5* was the most commonly affected gene, with a P/LP variant present in 43.6% of patients (Table 2). This might be due to the inclusion of older results (15 years), where less sensitive methods were used for genetic testing and the bias was expected. This group had the lowest age at diagnosis, indicating that patients with *COL4A5* variants present with kidney involvement early and are predicted to have a poorer prognosis than children with *COL4A3* and *COL4A4* variants. The study by Gibson et al. revealed that nearly 50% of Europeans with a predicted pathogenic *COL4A5* variant possessed the p.(Gly624Asp) variant, which was absent in other ancestries [17]. This variant was also the most prevalent in our cohort. Variants in *COL4A4* (40.0% of patients) and *COL4A3* (16.4% of patients) followed according to frequency. Among patients with autosomal AKD, we identified 40 heterozygotes, five heterozygotes with an additional VUS reported, and two compound heterozygotes. No homozygous patients were detected. Since frequencies of LP/P variants are extremely low in general populations, homozygous variants are not to be expected (except in isolated communities, which is generally not the case in our population). Similarly to other reports in the literature [4, 7, 8], heterozygous patients in our study were not completely asymptomatic but presented with renal and extrarenal manifestations. Hearing loss was reported in 6.7% of autosomal heterozygotes, consistent with the already published data [7]. Digenic AKD refers to the inheritance of two P/LP variants in *COL4A3–5*, each in a different gene [10, 22]. In our cohort, we confirmed one digenic AKD, with variants in *COL4A3* and *COL4A4*. Other combinations were found but could not count as digenic at the time of our study, since a VUS was reported in the second gene. Based on population studies, digenic disease with a pathogenic variant in *COL4A3* plus a pathogenic variant in *COL4A4* is more common than a pathogenic variant in COL4A5 plus one in *COL4A3* or *COL4A4*, because pathogenic *COL4A3* and *COL4A4* variants are more common [17]. The latter was also observed in our study.

Understanding genotype–phenotype correlations in AKD is important because they help predict the likely age of onset of kidney failure and the need for early and aggressive management with renin–angiotensin system blockade and other therapies [6]. Genotype–phenotype correlations also help to standardize the selection of patients with AKD for clinical treatment trials. Therefore, searching for novel variants and associating them with specific manifestations is important. Our study identified eight novel variants, one in *COL4A3*, three in *COL4A4*, and four in *COL4A5* gene, again demonstrating the increasing number of clinically important variants in *COL4A3–5* genes with further genetic development. Two of these variants are classified as VUS, indicating the need for further follow-up and re-evaluation. The phenotype of pediatric patients with newly described variants did not differ significantly in the presentation (Table 4). All patients had hematuria with a preserved kidney function and three had coexisting proteinuria. No one had proven ear or eye involvement. Nevertheless, the phenotypic severity varies among different variants, as observed in our cohort. The search for novel variants and their association with clinical manifestations is thus of significant clinical and scientific interest, as demonstrated by several recent discoveries [23, 24, 25].

Genetic testing is critical in the pediatric population, because timely and early genetic diagnosis of AKD in patients with glomerular hematuria allows for appropriate monitoring and pharmacologic intervention, which has a positive effect on kidney outcomes. The identification of a pathogenic variant in an index case also makes it easier to evaluate at-risk family members, as well as provide precise reproductive counseling and living-related donor kidney evaluation. Moreover, despite the typically mild phenotype in the initial years, a poorer prognosis was found in certain genotypes and initiating ACE therapy prior to the emergence of proteinuria is recommended in the guidelines [7, 10]. On the other hand, some children present with a more severe clinical picture from an early age, as described in the two clinical cases included above. Therefore, it is important to understand that children with AKD exhibit symptoms ranging from mild hematuria to kidney failure.

The limitations of our study include its retrospective nature and focus solely on the pediatric population. This limitation resulted in a lack of longer follow-up time to observe the complete clinical progression of the disease, which is essential for establishing a better genotype–phenotype correlation. Our study included only children and adolescents with genetically confirmed AKD, resulting in the exclusion of those with negative genetic results but positive biopsy findings and clinical symptoms consistent with AKD. This highlights the current limitations of genetic testing, which need to be addressed by further advances in the field. Future studies with prospective follow-up into adulthood are needed for a more comprehensive understanding of genotype–phenotype correlations.

## Conclusions

Our study reveals a diverse genetic background for AKD, with most cases presenting mild clinical symptoms during childhood and adolescence. However, a few exceptions noted in our study, along with data from the literature, highlight the critical importance of genetic analysis, confirmation of diagnosis, and patient risk stratification. These steps are essential for prompt treatment in childhood to delay kidney failure and extrarenal complications. Additionally, lifestyle risks and career choices can be more easily adjusted in children and should be guided by patient risk stratification after thorough counseling. Despite the current challenges in genetic evaluation, the emerging reclassification of AKD underscores the need for further investigation and correlation between the genotype and phenotype of pediatric patients with variants in *COL4A3*–*5* genes.

## Supplementary Information

Below is the link to the electronic supplementary material.

Graphical abstract (PPTX 109 KB)

## Data Availability

The data that supports the findings of this study are available from the corresponding author ABN, upon reasonable request.
