# Supplementary material for: A 15-year experience highlighting the spectrum of Alport kidney disease in the pediatric population and novel genetic variants in COL4A3–5
Source: Pediatr Nephrol. 2025 Feb 5;40(7):2215–23. doi: 10.1007/s00467-025-06683-8 (PMC12116809; doi:10.1007/s00467-025-06683-8)
Supplement: Supplementary file 1 — Graphical abstract (PPTX 109 KB) [file 467_2025_6683_MOESM1_ESM.pptx]

## Slide 1
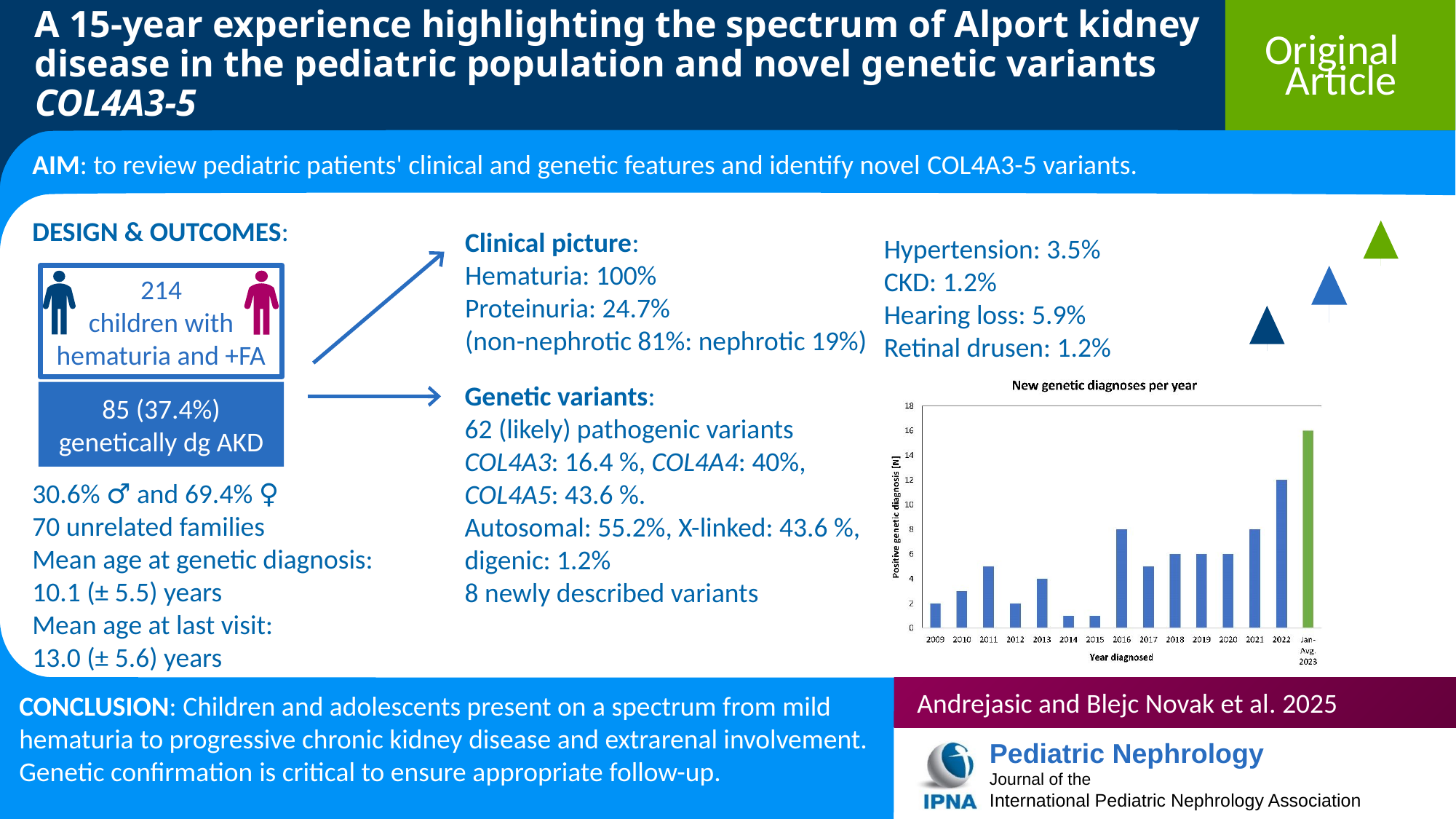

A 15-year experience highlighting the spectrum of Alport kidney disease in the pediatric population and novel genetic variants COL4A3-5
AIM: to review pediatric patients' clinical and genetic features and identify novel COL4A3-5 variants.
DESIGN & OUTCOMES:
30.6% ♂ and 69.4% ♀
70 unrelated families
Mean age at genetic diagnosis:
10.1 (± 5.5) years
Mean age at last visit:
13.0 (± 5.6) years
Clinical picture:
Hematuria: 100%
Proteinuria: 24.7%
(non-nephrotic 81%: nephrotic 19%)
Hypertension: 3.5%
CKD: 1.2%
Hearing loss: 5.9%
Retinal drusen: 1.2%
214
children with hematuria and +FA
Genetic variants:
62 (likely) pathogenic variants
COL4A3: 16.4 %, COL4A4: 40%,
COL4A5: 43.6 %.
Autosomal: 55.2%, X-linked: 43.6 %, digenic: 1.2%
8 newly described variants
85 (37.4%) genetically dg AKD
Andrejasic and Blejc Novak et al. 2025
CONCLUSION: Children and adolescents present on a spectrum from mild hematuria to progressive chronic kidney disease and extrarenal involvement. Genetic confirmation is critical to ensure appropriate follow-up.
